# Supplementary material for: Duplication, Loss, and Evolutionary Features of Specific UDP-Glucuronosyltransferase Genes in Carnivora (Mammalia, Laurasiatheria)
Source: Animals (Basel). 2022 Oct 27;12(21):2954. doi: 10.3390/ani12212954 (PMC9658400; doi:10.3390/ani12212954)
Supplement: Supplementary file 1 [file animals-12-02954-s001.zip › Supplemental Figure S1.pdf]

Supplemental Figure S1

Sequence nucleotide number in pinniuped UGT1A7-12s	565	566	567	568	569	570	571	572	573	574	575	576	577	578	579
Protein translated from codons in Human UGT1A9		A			R			G			I			L	
H. sapiens UGT1A9 NM 021027	G	C	C	A	G	G	G	G	A	A	T	A	C	T	T
N. vison UGT1A7-11 XM 044241893 LOC122902436	A	C	C	A	G	G	G	G	A	G	T	A	T	T	T
C. lupus familiaris UGT1A7-12 XM 038434521	T	T	G	C	G	G	A	A	C	A	T	T	C	C	A
M. musculus Ugt1a9 NM 201644	G	C	A	A	G	A	G	G	T	G	T	A	T	T	T
C. ursinus UGT1A7-12 LOC112836383 XM 025888034	A	C	T	A	G	G	G	G	A	G	T	A	T	T	T
Z. californianus UGT1A7-12 LOC113919635 XM 035726859	C	C	C	A	G	G	G	G	A	G	T	A	T	T	T
Protein translated from codons in Z. californianus UGT1A7-12		P			R			G			V			F	

Sequence nucleotide number in pinniuped UGT1A7-12s	580	581	582	583	584	585	586	587	588	589	590	591	592	593	594
Protein translated from codons in Human UGT1A9		C			H			Y			L			E	
H. sapiens UGT1A9 NM 021027	T	G	C	C	A	C	T	A	T	C	T	T	G	A	A
N. vison UGT1A7-11 XM 044241893 LOC122902436	T	G	T	C	A	T	T	A	T	C	T	T	G	A	A
C. lupus familiaris UGT1A7-12 XM 038434521	T	G	T	G	A	T	T	T	A	G	A	A	T	C	T
M. musculus Ugt1a9 NM 201644	T	G	T	G	A	C	T	A	T	C	T	T	G	A	A
C. ursinus UGT1A7-12 LOC112836383 XM 025888034	T	G	C	C	A	T	T	A	G	C	T	T	G	A	A
Z. californianus UGT1A7-12 LOC113919635 XM 035726859	T	G	C	C	A	T	T	A	G	C	T	T	G	A	A
Protein translated from codons in Z. californianus UGT1A7-12		C			H			*			L			E	

Sequence nucleotide number in pinniuped UGT1A7-12s	595	596	597	598	598	598	598	598	598	598	598	598	598	598	598
Protein translated from codons in Human UGT1A9		E			G			A			Q			C	
H. sapiens UGT1A9 NM 021027	G	A	A	G	G	T	G	C	A	C	A	G	T	G	C
N. vison UGT1A7-11 XM 044241893 LOC122902436	G	A	A	G	G	A	G	C	A	C	A	G	T	G	C
C. lupus familiaris UGT1A7-12 XM 038434521	G	A	G	A	G	C	A	C	G	C	A	G	T	G	C
M. musculus Ugt1a9 NM 201644	G	A	G	G	G	T	G	C	C	C	A	G	T	G	C
C. ursinus UGT1A7-12 LOC112836383 XM 025888034	G	A	A	G	G	C	A	C	A	C	A	G	T	G	T
Z. californianus UGT1A7-12 LOC113919635 XM 035726859	G	A	A	G	G	C	A	C	A	C	A	G	T	G	T
Protein translated from codons in Z. californianus UGT1A7-12		E			G			T			Q			C	
