# Supplementary material for: Duplication, Loss, and Evolutionary Features of Specific UDP-Glucuronosyltransferase Genes in Carnivora (Mammalia, Laurasiatheria)
Source: Animals (Basel). 2022 Oct 27;12(21):2954. doi: 10.3390/ani12212954 (PMC9658400; doi:10.3390/ani12212954)
Supplement: Supplementary file 1 [file animals-12-02954-s001.zip › Supplemental Table S1.pdf]

Supplemental Data

Supplemental Table S1: Foraging habitat of Carnivora

Species	Food habitats	References
Acinonyx jubatus	Carnivore	[1,2]
Ailuropoda melanoleuca	Herbivore	[3,4]
Callorhinus ursinus	Fishivore	[5–8]
Canis lupus familiaris	Omnivore	[9–11]
Crocuta crocuta	Insectivore, Omnivore	[12,13]
Enhydra lutris kenyoni	Fishivore	[8,14]
Eumetopias jubatus	Fishivore	[8,15]
Felis catus	Carnivora	[16,17]
Halichoerus grypus	Fishivore	[18]
Hyaena hyaena	Carnivore	[19,20]
Leptonychotes weddellii	Fishivore	[8,21]
Lontra canadensis	Fishivore	[22,23]
Lutra lutra	Fishivore	[24]
Lynx canadensis	Carnivora	[25,26]
Meles meles	Insectivore, Omnivore	[27,28]
Mirounga angustirostris	Fishivore	[29,30]
Mirounga leonina	Fishivore	[31,32]
Mustela erminea	Carnivora	[33,34]
Mustela putorius furo	Carnivora	[35,36]
Neogale vison	Carnivora	[37–39]
Neomonachus schauinslandi	Fishivore	[8,40]
Odobenus rosmarus divergens	Fishivore	[41,42]
Panthera leo	Carnivora	[43,44]
Panthera pardus	Carnivora	[45,46]
Panthera tigris	Carnivora	[47,48]
Phoca vitulina	Fishivore	[8,49]
Prionailurus bengalensis	Carnivora	[50,51]
Prionailurus viverrinus	Carnivora	[50]
Puma concolor	Carnivora	[52,53]

Puma yagouaroundi	**Carnivora**	[54,55]
Ursus americanus	**Omnivore**	[56–59]
Ursus arctos	**Omnivore**	[60–62]
Ursus maritimus	**Carnivore**	[61,63]
Vulpes lagopus	**Omnivore**	[64,65]
Vulpes vulpes	**Omnivore**	[66,67]
Zalophus californianus	**Fishivore**	[68,69]

1. Rezaei Khozani, A.; Kaboli, M.; Ashrafi, S.; Akbari, H. Survey Diet of Asiatic Cheetah Acinonyx Jubatus Venaticus by Scat Analysis Method in Bafgh Protected Area, Central Iran. *J. Anim. Environ.* **2016**, *8*, 1–8.
2. Mills, M.G.L.; Broomhall, L.S.; Du Toit, J.T. Cheetah Acinonyx Jubatus Feeding Ecology in the Kruger National Park and a Comparison across African Savanna Habitats: Is the Cheetah Only a Successful Hunter on Open Grassland Plains? *Wildlife Biol.* **2004**, *10*, 177–186, doi:10.2981/WLB.2004.024.
3. Wei, F.; Hu, Y.; Yan, L.; Nie, Y.; Wu, Q.; Zhang, Z. Giant Pandas Are Not an Evolutionary Cul-de-Sac: Evidence from Multidisciplinary Research. *Mol. Biol. Evol.* **2015**, *32*, 4–12, doi:10.1093/MOLBEV/MSU278.
4. Schaller, G.B. The Giant Pandas of Wolong. **1985**, 298.
5. Gudmundson, C.J.; Zeppelin, T.K.; Ream, R.R. Application of Two Methods for Determining Diet of Northern Fur Seals (Callorhinus Ursinus). *Fish. Bull.* **2006**, *104*, 445–456.
6. Berta, A.; Churchill, M.; Boessenecker, R.W. The Origin and Evolutionary Biology of Pinnipeds: Seals, Sea Lions, and Walruses. *Annu. Rev. Earth Planet. Sci.* **2018**, *46*, 203–228, doi:10.1146/annurev-earth-082517-010009.
7. Ethology and Behavioral Ecology of Phocids. **2022**, doi:10.1007/978-3-030-88923-4.

8. Pauly, D.; Trites, A.W.; Capuli, E.; Christensen, V. Diet Composition and Trophic Levels of Marine Mammals. *ICES J. Mar. Sci.* **1998**, *55*, 467–481, doi:10.1006/JMSC.1997.0280.

9. Bosch, G.; Hagen-Plantinga, E.A.; Hendriks, W.H. Dietary Nutrient Profiles of Wild Wolves: Insights for Optimal Dog Nutrition? *Br. J. Nutr.* **2015**, *113*, S40–S54, doi:10.1017/S0007114514002311.

10. Soars, M.G.M.; Riley, R.J.R.R.J.; Findlay, K.A.B.K.K.A.B.; Coffey, M.J.; Burchell, B.; … M.C.-D.M. and; 2001, U.; Coffey, M.J.; Burchell, B. Evidence for Significant Differences in Microsomal Drug Glucuronidation by Canine and Human Liver and Kidney. *Drug Metab. Dispos.* **2001**, *29*, 121–126.

11. Baker, D.H.; Czarnecki-Maulden, G.L. Comparative Nutrition of Cats and Dogs. *Annu. Rev. Nutr.* **1991**, *11*, 239–263, doi:10.1146/ANNUREV.NU.11.070191.001323.

12. Montanari, S. Discrimination Factors of Carbon and Nitrogen Stable Isotopes in Meerkat Feces. *PeerJ* **2017**, *2017*, e3436, doi:10.7717/PEERJ.3436.

13. Brox, B.W.; Edwards, K.; Buist, N.A.; Macaskill, A.C. Investigating Food Preference in Zoo-Housed Meerkats. *Zoo Biol.* **2021**, *40*, 517–526, doi:10.1002/ZOO.21640.

14. Johnson, C.K.; Tinker, M.T.; Estes, J.A.; Conrad, P.A.; Staedler, M.; Miller, M.A.; Jessup, D.A.; Mazet, J.A.K. Prey Choice and Habitat Use Drive Sea Otter Pathogen Exposure in a Resource-Limited Coastal System. *Proc. Natl. Acad. Sci. U. S. A.* **2009**, *106*, 2242–2247, doi:10.1073/PNAS.0806449106/SUPPL_FILE/0806449106SI.PDF.

15. Rand, K.; McDermott, S.; Logerwell, E.; Matta, M.E.; Levine, M.; Bryan, D.R.; Spies, I.B.; Loomis, T. Higher Aggregation of Key Prey Species Associated with Diet and Abundance of the Steller Sea Lion *Eumetopias Jubatus* across the Aleutian Islands. *Mar. Coast. Fish.* **2019**, *11*, 472–486, doi:10.1002/MCF2.10096--.

16. Edinboro, C.H.; Scott-Moncrieff, J.C.; Janovitz, E.; Thacker, H.L.; Glickman, L.T. Epidemiologic Study of Relationships between Consumption of Commercial Canned Food and Risk of Hyperthyroidism in Cats. *J. Am. Vet. Med. Assoc.* **2004**, *224*, 879–886, doi:10.2460/javma.2004.224.879.

17. Davies, M. Veterinary Clinical Nutrition: Success Stories: An Overview. *Proc. Nutr. Soc.* **2016**, *75*, 392–397, doi:10.1017/S002966511600029X.

18. Hammill, M.O.; Stenson, G.B. Estimated Prey Consumption by Harp Seals (Phoca Groenlandica) & Hooded Seals (Cystophora Cristata) & Grey Seals (Halichoerus Grypus) and Harbour Seals (Phoca Vitulina) in Atlantic Canada. *J. Northw. Atl. Fish. Sci* **2000**, *26*, 1–23.

19. Alam, M.S.; Khan, J.A. Food Habits of Striped Hyena (Hyaena Hyaena) in a Semi-Arid Conservation Area of India. *J. Arid L. 2015 76* **2015**, *7*, 860–866, doi:10.1007/S40333-015-0007-2.

20. Bhandari, S.; Morley, C.; Aryal, A.; Shrestha, U.B. The Diet of the Striped Hyena in Nepal's Lowland Regions. *Ecol. Evol.* **2020**, *10*, 7953–7962, doi:10.1002/ECE3.6223.

21. Goetz, K.T.; Burns, J.M.; Hückstädt, L.A.; Shero, M.R.; Costa, D.P. Temporal Variation in Isotopic Composition and Diet of Weddell Seals in the Western Ross Sea. *Deep. Res. Part II Top. Stud. Oceanogr.* **2017**, *140*, 36–44, doi:10.1016/J.DSR2.2016.05.017.

22. Cote, D.; Stewart, H.M.J.; Gregory, R.S.; Gosse, J.; Reynolds, J.J.; Stenson, G.B.; Miller, E.H. Prey Selection by Marine-Coastal River Otters (Lontra Canadensis) in Newfoundland, Canada. *J. Mammal.* **2008**, *89*, 1001–1011, doi:10.1644/07-MAMM-A-192.1.

23. Day, C.C.; Westover, M.D.; McMillan, B.R. Seasonal Diet of the Northern River Otter (Lontra Canadensis): What Drives Prey Selection? *https://doi.org/10.1139/cjz-2014-0218* **2015**, *93*, 197–205, doi:10.1139/CJZ-2014-0218.

24. Krawczyk, A.J.; Bogdziewicz, M.; Majkowska, K.; Glazaczow, A. Diet Composition of the Eurasian Otter Lutra Lutra in Different Freshwater Habitats of Temperate Europe: A Review and Meta-Analysis. *Mamm. Rev.* **2016**, *46*, 106–113, doi:10.1111/MAM.12054/SUPPINFO.

25. Burstahler, C.M.; Terwissen, C. V.; Roth, J.D. Latitudinal Gradient in Cortisol Concentrations in Canada Lynx (Lynx Canadensis) Is Not Explained by Diet. *Can. J. Zool.* **2019**, *97*, 748–753, doi:10.1139/CJZ-2018-0204/SUPPL_FILE/CJZ-2018-0204SUPPLA.DOCX.

26. Parker, G.R.; Maxwell, J.W.; Morton, L.D.; Smith, G.E.J. The Ecology of the Lynx (Lynx Canadensis) on Cape Breton Island. *https://doi.org/10.1139/z83-102* **2011**, *61*, 770–786, doi:10.1139/Z83-102.

27. Balestrieri, A.; Remonti, L.; Saino, N.; Raubenheimer, D. The 'Omnivorous Badger Dilemma': Towards an Integration of Nutrition with the Dietary Niche in Wild Mammals. *Mamm. Rev.* **2019**, *49*, 324–339, doi:10.1111/MAM.12164.

28. Cleary, G.P.; Corner, L.A.L.; O'Keeffe, J.; Marples, N.M. The Diet of the Badger Meles Meles in the Republic of Ireland. *Mamm. Biol. 2009 746* **2009**, *74*, 438–447, doi:10.1016/J.MAMBIO.2009.07.003.

29. Costa, D.P. (Daniel P.; McHuron, E.A. Ethology and Behavioral Ecology of Phocids. 645.

30. Rita, D.; Drago, M.; Galimberti, F.; Cardona, L. Temporal Consistency of Individual Trophic Specialization in Southern Elephant Seals Mirounga Leonina. *Mar. Ecol. Prog. Ser.* **2017**, *585*, 229–242, doi:10.3354/MEPS12411.

31. Spurlin, S.M.; Peterson, S.H.; Crocker, D.E.; Costa, D.P. Nitrogen and Carbon Stable-Isotope Ratios Change in Adult Northern Elephant Seals (Mirounga Angustirostris) during the Breeding and Molting Fasts. *Mar. Mammal Sci.* **2019**, *35*, 707–717, doi:10.1111/MMS.12558.
32. Condit, R.; Le Boeuf, B.J. Feeding Habits and Feeding Grounds of the Northern Elephant Seal. *J. Mammal.* **1984**, *65*, 281–290, doi:10.2307/1381167.
33. Dell'Arte, G.L.; Laaksonen, T.; Norrdahl, K.; Korpimäki, E. Variation in the Diet Composition of a Generalist Predator, the Red Fox, in Relation to Season and Density of Main Prey. *Acta Oecologica* **2007**, *31*, 276–281, doi:10.1016/J.ACTAO.2006.12.007.
34. McDonald, R.A.; Webbon, C.; Harris, S. The Diet of Stoats (Mustela Erminea) and Weasels (Mustela Nivalis) in Great Britain. *J. Zool.* **2000**, *252*, 363–371, doi:10.1111/J.1469-7998.2000.TB00631.X.
35. Smith, G.P.; Ragg, J.R.; Waldrup, K.A.; Moller, H. Diet of Feral Ferrets (Mustela Furo) from Pastoral Habitats in Otago and Southland, New Zealand. *New Zeal. J. Zool.* **1995**, *22*, 363–369, doi:10.1080/03014223.1995.9518054.
36. Vinke, C.M.; Schoemaker, N.J. The Welfare of Ferrets (Mustela Putorius Furo T): A Review on the Housing and Management of Pet Ferrets. *Appl. Anim. Behav. Sci.* **2012**, *139*, 155–168, doi:10.1016/J.APPLANIM.2012.03.016.
37. Huang, A.C.; Nelson, C.; Elliott, J.E.; Guertin, D.A.; Ritland, C.; Drouillard, K.; Cheng, K.M.; Schwantje, H.M. River Otters (Lontra Canadensis) "Trapped" in a Coastal Environment Contaminated with Persistent Organic Pollutants: Demographic and Physiological Consequences. *Environ. Pollut.* **2018**, *238*, 306–316, doi:10.1016/j.envpol.2018.03.035.

38. Krawczyk, A.J.; Bogdziewicz, M.; Czyz, M.J. Diet of the American Mink Neovison Vison in an Agricultural Landscape in Western Poland. *https://doi.org/10.25225/fozo.v62.i4.a8.2013* **2013**, *62*, 304–310, doi:10.25225/FOZO.V62.I4.A8.2013.

39. Magnusdottir, R.; von Schmalensee, M.; Stefansson, R.A.; Macdonald, D.W.; Hersteinsson, P. A Foe in Woe: American Mink (Neovison Vison) Diet Changes during a Population Decrease. *Mamm. Biol. 2014 791* **2013**, *79*, 58–63, doi:10.1016/J.MAMBIO.2013.08.002.

40. Robinson, S.; Barbieri, M.; Johanos, T. The Hawaiian Monk Seal: Ethology Applied to Endangered Species Conservation and Recovery. **2022**, 599–635, doi:10.1007/978-3-030-88923-4_16.

41. Sheffield, G.; Grebmeier, J.M. Pacific Walrus (Odobenus Rosmarus Divergens):</Br>Differential Prey Digestion and Diet. *Mar. Mammal Sci.* **2009**, *25*, 761–777, doi:10.1111/J.1748-7692.2009.00316.X.

42. Clark, C.T.; Horstmann, L.; Vernal, A. de; Jensen, A.M.; Misarti, N. Pacific Walrus Diet across 4000 Years of Changing Sea Ice Conditions. *Quat. Res.* **2022**, *108*, 26–42, doi:10.1017/QUA.2018.140.

43. Beukes, M.; Radloff, F.G.T.; Ferreira, S.M. Estimating Lion's Prey Species Profile in an Arid Environment. *J. Zool.* **2017**, *303*, 136–144, doi:10.1111/JZO.12474.

44. Bothma, J. du P.; Walker, C. The African Lion. *Larg. Carniv. African Savannas* **1999**, 22–59, doi:10.1007/978-3-662-03766-9_2/COVER.

45. Kshettry, A.; Vaidyanathan, S.; Athreya, V. Diet Selection of Leopards (Panthera Pardus) in a Human-Use Landscape in North-Eastern India: *https://doi.org/10.1177/1940082918764635* **2018**, *11*, doi:10.1177/1940082918764635.

46. Andheria, A.P.; Karanth, K.U.; Kumar, N.S. Diet and Prey Profiles of Three Sympatric Large Carnivores in Bandipur Tiger Reserve, India. *J. Zool.* **2007**, *273*, 169–175, doi:10.1111/J.1469-7998.2007.00310.X.

47. Hayward, M.W.; Jedrzejewski, W.; Jedrzewska, B. Prey Preferences of the Tiger P Anthera Tigris. *J. Zool.* **2012**, *286*, 221–231, doi:10.1111/J.1469-7998.2011.00871.X.

48. Biswas, S.; Sankar, K. Prey Abundance and Food Habit of Tigers (Panthera Tigris Tigris) in Pench National Park, Madhya Pradesh, India. *J. Zool.* **2002**, *256*, 411–420, doi:10.1017/S0952836902000456.

49. Toth, J.; Evert, S.; Zimmermann, E.; Sullivan, M.; Dotts, L.; Able, K.W.; Hagan, R.; Slocum, C. Annual Residency Patterns and Diet of Phoca Vitulina Concolor (Western Atlantic Harbor Seal) in a Southern New Jersey Estuary. *https://doi.org/10.1656/045.025.0407* **2018**, *25*, 611–626, doi:10.1656/045.025.0407.

50. Ganguly, D.; Adhya, T. How Fishing Cats Prionailurus Viverrinus Bennett, 1833 Fish: Describing a Felid's Strategy to Hunt Aquatic Prey. *Mammalia* **2022**, *86*, 182–189, doi:10.1515/MAMMALIA-2020-0133/DOWNLOADASSET/SUPPL/J_MAMMALIA-2020-0133_SUPPL_003.JPG.

51. Grassman, L.I.; Tewes, M.E.; Silvy, N.J.; Kreetiyutanont, K. Spatial Organization and Diet of the Leopard Cat (Prionailurus Bengalensis) in North-Central Thailand. *J. Zool.* **2005**, *266*, 45–54, doi:10.1017/S095283690500659X.

52. Rau, J.R.; Jiménez, J.E. Diet of Puma (Puma Concolor, Carnivora: Felidae) in Coastal and Andean Ranges of Southern Chile. *Stud. Neotrop. Fauna Environ.* **2002**, *37*, 201–205, doi:10.1076/SNFE.37.3.201.8567.

53. Aranda, M.; Sánchez-Cordero, V. Prey Spectra of Jaguar (Panthera Onca) and Puma (Puma Concolor) in Tropical Forests of Mexico. *Stud. Neotrop. Fauna Environ.* **1996**, *31*, 65–67, doi:10.1076/SNFE.31.2.65.13334.
54. Tófoli, C.F.; Rohe, F.; Setz, E.Z.F. Jaguarundi (Puma Yagouaroundi) (Geoffroy, 1803) (Carnivora, Felidae) Food Habits in a Mosaic of Atlantic Rainforest and Eucalypt Plantations of Southeastern Brazil. *Brazilian J. Biol.* **2009**, *69*, 871–877, doi:10.1590/S1519-69842009000400015.
55. Bianchi, R. de C.; Rosa, A.F.; Gatti, A.; Mendes, S.L. Diet of Margay, Leopardus Wiedii, and Jaguarundi, Puma Yagouaroundi, (Carnivora: Felidae) in Atlantic Rainforest, Brazil. *Zool.* **2011**, *28*, 127–132, doi:10.1590/S1984-46702011000100018.
56. Costello, C.M.; Cain, S.L.; Pils, S.; Frattaroli, L.; Haroldson, M.A.; Van Manen, F.T. Diet and Macronutrient Optimization in Wild Ursids: A Comparison of Grizzly Bears with Sympatric and Allopatric Black Bears. *PLoS One* **2016**, *11*, doi:10.1371/JOURNAL.PONE.0153702.
57. Merkle, J.A.; Polfus, J.L.; Derbridge, J.J.; Heinemeyer, K.S. Dietary Niche Partitioning among Black Bears, Grizzly Bears, and Wolves in a Multiprey Ecosystem. *Can. J. Zool.* **2017**, *95*, 663–671, doi:10.1139/CJZ-2016-0258.
58. Kirby, R.; Alldredge, M.W.; Pauli, J.N. The Diet of Black Bears Tracks the Human Footprint across a Rapidly Developing Landscape. *Biol. Conserv.* **2016**, *200*, 51–59, doi:10.1016/J.BIOCON.2016.05.012.
59. Hatch, K.A.; Kester, K.A.; Auger, J.; Roeder, B.L.; Bunnell, K.; Black, H.L. The Effect of Sex, Age, and Location on Carnivory in Utah Black Bears (Ursus Americanus). *Oecologia* **2019**, *189*, 931–937, doi:10.1007/S00442-019-04385-1.

60. Matsubayashi, J.; Otsubo, K.; Morimoto, J.O.; Nakamura, F.; Nose, T.; Tayasu, I. Feeding Habits May Explain the Morphological Uniqueness of Brown Bears on Etorofu Island, Southern Kuril Islands in East Asia. *Biol. J. Linn. Soc.* **2016**, *119*, 99–105, doi:10.1111/BIJ.12798.

61. Mangipane, L.S.; Lafferty, D.J.R.; Joly, K.; Sorum, M.S.; Cameron, M.D.; Belant, J.L.; Hilderbrand, G. V.; Gustine, D.D. Dietary Plasticity and the Importance of Salmon to Brown Bear (Ursus Arctos) Body Size and Condition in a Low Arctic Ecosystem. *Polar Biol.* **2020**, *43*, 825–833, doi:10.1007/S00300-020-02690-7.

62. Mangipane, L.S.; Belant, J.L.; Lafferty, D.J.R.; Gustine, D.D.; Hiller, T.L.; Colvin, M.E.; Mangipane, B.A.; Hilderbrand, G. V. Dietary Plasticity in a Nutrient-Rich System Does Not Influence Brown Bear (Ursus Arctos) Body Condition or Denning. *Polar Biol.* **2018**, *41*, 763–772, doi:10.1007/S00300-017-2237-6.

63. Johnson, A.C.; Hobson, K.A.; Lunn, N.J.; McGeachy, D.; Richardson, E.S.; Derocher, A.E. Temporal and Intra-Population Patterns in Polar Bear Foraging Ecology in Western Hudson Bay. *Mar. Ecol. Prog. Ser.* **2019**, *619*, 187–199, doi:10.3354/MEPS12933.

64. Elmhagen, B.; Tannerfeldt, M.; Verucci, P.; Angerbjörn, A. The Arctic Fox (Alopex Lagopus): An Opportunistic Specialist . *J. Zool.* **2000**, *251*, 139–149, doi:10.1111/J.1469-7998.2000.TB00599.X.

65. Food Habits of Arctic Foxes (Alopex Lagopus) on the Western Coast of Svalbard on JSTOR Available online: https://www.jstor.org/stable/40511362 (accessed on 31 July 2022).

66. Baltrūnaitė, L. Diet and Winter Habitat Use of the Red Fox, Pine Marten and Raccoon Dog in Dzūkija National Park, Lithuania. *Acta Zool. Litu.* **2006**, *16*, 46–53, doi:10.1080/13921657.2006.10512709.

67. Castañeda, I.; Doherty, T.S.; Fleming, P.A.; Stobo-Wilson, A.M.; Woinarski, J.C.Z.; Newsome, T.M. Variation in Red Fox Vulpes Vulpes Diet in Five Continents. *Mamm. Rev.* **2022**, *52*, 328–342, doi:10.1111/MAM.12292.
68. Weise, M.J.; Harvey, J.T. Temporal Variability in Ocean Climate and California Sea Lion Diet and Biomass Consumption: Implications for Fisheries Management. *Mar. Ecol. Prog. Ser.* **2008**, *373*, 157–172, doi:10.3354/MEPS07737.
69. Bailey, K.M.; Ainley, D.G. The Dynamics of California Sea Lion Predation on Pacific Hake. *Fish. Res.* **1981**, *1*, 163–176, doi:10.1016/0165-7836(81)90018-7.
